# Modern Surgical Approaches to Pulmonary Hydatidosis

**DOI:** 10.4269/ajtmh.25-0251

**Published:** 2025-06-17

**Authors:** Anne Spichler-Moffarah, Joseph M. Vinetz

**Affiliations:** Section of Infectious Diseases, Department of Internal Medicine, Yale School of Medicine, New Haven, Connecticut

*Echinococcus granulosus*, a cestode parasite, causes hydatid disease or cystic echinococcosis (CE), which most commonly involves the liver, and less commonly, the lungs or other organs.^1^ The disease is found worldwide with notable prevalence in southern and eastern Europe, South America, and parts of Asia, including the Middle East.^2^ *E. granulosus* is transmitted in the United States, as previously reported in this journal.^3^^,^^4^ The lungs account for approximately 20% of reported CE cases.^5^ Canids, in which intestinal *E. granulosus* tapeworms develop, are the definitive hosts, leading to shedding of eggs that humans, as accidental hosts, ingest. The eggs develop into oncospheres, which invade and disseminate to target organs. In target organs, oncospheres transform and enlarge concentrically into fluid- and protoscolex-filled cysts. These cysts start as unilocular structures and later undergo septation. Cysts consist of an inner germinal and nucleated syncytial layer supported externally by a carbohydrate-rich acellular laminated layer of variable thickness, surrounded by a host-produced fibrous adventitial layer. Daughter cysts may develop within large primary cysts or from protoscolices that arise from ruptured primary cysts.^2^^,^^6^ Cysts may die and calcify, remain *in situ, *or enlarge, and they usually become symptomatic due to mass effect or rupture. Primary pulmonary hydatid cysts are embedded in lung parenchyma without pronounced tissue reaction, in contrast to liver cysts. With cyst rupture, clinical consequences include allergic responses such anaphylaxis and hives; later development of bronchopleural fistula can occur, with secondary pleural empyema.

Pulmonary hydatid cysts, which may not be suspected without a suitable travel or exposure history, often remain silent for long periods before enlargement causes symptoms or they are noted as incidental findings on chest radiography. Common manifestations include production of salty fluid in the sputum (“vomica”), chest pain, cough, and hemoptysis; other clinical features may include compression or damage to bronchi, blood vessels, the rib cage, or mediastinal organs. When cysts rupture, fever, urticaria, eosinophilia, anaphylactic shock, cyst dissemination, and cyst-bronchial fistulas may occur.^6^^,^^7^ Once the diagnosis is suspected on the basis of chest x-ray and history, more advanced imaging such as ultrasound, computed tomography, and magnetic resonance imaging may be useful. Serology is a confirmatory step, with various levels of sensitivity and specificity corresponding to lesion location and how the ELISA antigen is prepared.^2^^,^^6^ A uniform treatment regimen or approach may not be feasible because of the variability of pulmonary echinococcosis, but management includes surgical and antiparasitic drugs, primarily albendazole and praziquantel, used singly or in combination.^2^^,^^5^

Multiple surgical modalities for treatment of pulmonary echinococcus have been developed, depending on cyst characteristics (calcified or not), location, and complications.^5^^,^^7^ Cure is achieved by the complete removal of the cyst (enucleation). Open thoracotomy has been the preferred surgical approach because of concern regarding the risk of intraoperative cyst rupture. Median sternotomy is considered useful for the surgical treatment of bilateral lung hydatid cysts. Percutaneous aspiration, instillation of scolicidal agents, and re-aspiration of contents (PAIR) has largely been disappointing in lung hydatidosis.^8^ More recently, minimally invasive thoracoscopic techniques or video-assisted thoracoscopic surgery (VATS) have been shown to be safe therapeutic options, particularly for small and peripheral cysts. If the cyst cannot be removed in totality, including the adventitia, continued antiparasitic drugs may be necessary to keep the cyst from recurring, but duration of treatment is unclear.^2^

In this issue, Lissandrin and colleagues report an observational surgical study of 22 echinococcus cases involving the lung, including some cases with liver or other organ involvement.^9^ They report their experience using VATS and open thoracotomy for hydatid cyst removal. They recommend enucleation and residual cavity cleaning with hypertonic saline but favor the least aggressive approach to spare tissue damage, especially in younger patients. Inclusion criteria for the study were: at least one intrathoracic cyst; a subjective clinical need for surgery; confirmation of diagnosis by advanced radiographic imaging; and three different positive serological tests confirming the diagnosis of CE. They studied whether tissue sparing surgery was as effective as a more extensive thoracotomy. The subjects were mostly young adults without comorbidities or evidence of cyst rupture before the time of surgery. All patients received albendazole 24 hours before surgery, generally continuing for 1 month, postoperatively. Roughly half of the patients underwent VATS; the others underwent anterior thoracotomy based on clinical judgment. None of the patients required a lobectomy. Two had major complications, with the development of bronchopleural fistulae. Postoperative follow up was generally uneventful and follow up at almost 12 months did not show disease recurrence or mortality. The authors concluded that whenever possible, tissue-saving techniques should be prioritized to preserve lung function. One interesting suggestion was that albendazole, the standard antiparasitic, should be avoided preoperatively for larger cysts, with the explanation that treatment may cause detachment of the endocyst and lead to cyst bronchial fistula. Also, it was noted that cysts treated with a long presurgical course of albendazole were difficult to dissociate from surrounding tissue.^5^

The limitations of the study include small numbers of patients, lack of randomization, and non-standardized medical treatment regimens. However, the relative rarity of pulmonary echinococcosis makes a randomized, double-blinded trial comparing management approaches virtually impossible. Consideration of adjunctive medical treatment (in addition to surgery) versus primary medical treatment with antiparasitics alone was clearly missing from this study. Prospective studies of treatment strategies, considering albendazole vs. praziquantel versus a combination of the two, and schedules and duration of treatment, would be very helpful.

The important paper by Lissandrin et al. in this issue describes surgical approaches to pulmonary hydatid disease and outcomes in a carefully selected, small number of patients.^9^ This work brings to our attention important progress in the combined medical and surgical management of this uncommon parasitic disease, informing clinical practice globally. More data on the management of CE from larger prospective studies are welcome.
